# Analysis of neoadjuvant chemotherapy for breast cancer: a 20-year retrospective analysis of patients of a single institution

**DOI:** 10.1186/s12885-023-11505-x

**Published:** 2023-10-16

**Authors:** Danzhi Chen, Qinchuan Wang, Minjun Dong, Fei Chen, Aihua Huang, Cong Chen, Yi Lu, Wenhe Zhao, Linbo Wang

**Affiliations:** 1 Department of Surgical Oncology, Sir Run Run Shaw Hospital, Zhejiang University School of Medicine, 3 East Qingchun Road, Hangzhou, 310016 People’s Republic of China; 2 Department of Big Data and Health Statistics, School of Public Health, Zhejiang University School of Medicine, Hangzhou, China; 3 Department of Pathology, Sir Run Run Shaw Hospital, Zhejiang University School of Medicine, Hangzhou, China; 4 Department of Breast Surgery, Affiliated Hangzhou First People’s Hospital, Zhejiang University School of Medicine, Hangzhou, China; 5 Shaoxing Hospital, Shaoxing People’s Hospital, Zhejiang University School of Medicine, Shao, Xing China

## Abstract

**Background:**

Neoadjuvant chemotherapy (NAC) has been widely applied in operable breast cancer patients. This study aim to identify the predictive factors of overall survival(OS) and recurrence free survival (RFS) in breast cancer patients who received NAC from a single Chinese institution.

**Patients and Methods:**

There were 646 patients recruited in this study. All the patients were treated at department of Surgical Oncology, Sir Run Run Shaw Hospital between February 25, 1999 and August 22, 2018. The relevant clinicopathological and follow-up data were collected retrospectively. RFS and OS were assessed using the Kaplan-Meier method. Multivariate Cox proportional hazards model was also employed. Multi-variate logistic regression model was simulated to predict pathologic complete response (pCR).

**Results:**

In total, 118 patients (18.2%) achieved pCR during NAC. The 5-year OS was 94.6% versus 78.1% in patients with and without pCR, respectively (P < 0.001). The 5-year RFS was 95.3% and 72.7%, respectively (P < 0.001). No difference was detected among molecular subtypes of 5-year RFS in patients obtained pCR. Factors independently predicting RFS were HER2-positive subtype (hazard ratio(HR), 1.906; P = 0.004), triple-negative breast cancer (TNBC) (HR,2.079; P = 0.003), lymph node positive after NAC(HR,2.939; P < 0.001), pCR (HR, 0.396;P = 0.010), and clinical stage III (HR,2.950; P = 0.016). Multi-variate logistic regression model was simulated to predict the pCR rate after NAC, according to clinical stage, molecular subtype, ki-67, LVSI, treatment period and histology. In the ROC curve analysis, the AUC of the nomogram was 0.734 (95%CI,0.867–12.867).

**Conclusions:**

Following NAC, we found that pCR positively correlated with prognosis and the molecular subtype was a prognostic factor.

**Supplementary Information:**

The online version contains supplementary material available at 10.1186/s12885-023-11505-x.

## Introduction

Neoadjuvant chemotherapy (NAC) is increasingly being utilized as the frontline therapy for the management of high-risk early-stage breast cancer in operable patients [1, 2]. From a research perspective, neoadjuvant therapy was recognized as a human in vivo system for evaluating predictive biomarkers, alternative endpoints, and the therapeutic efficacy of including novel agents [3, 4]. Studies have demonstrated no difference in survival between adjuvant or neoadjuvant settings [1]. However, neoadjuvant therapy improves breast-conserving surgery success rates due to tumor downstaging [5–7] and allows for response assessment. Pathologic complete response (pCR) to neoadjuvant chemotherapy is associated with favorable long-term outcomes [8], however, most patients cannot achieve pCR in clinical practice. Whether pCR is accomplished or not, there are other factors that indicate poor prognosis in patients after NAC. The neoadjuvant therapy model provides a potentially efficient trial design to explore the efficacy of novel therapies utilizing pCR as a surrogate marker for disease-free survival (DFS) and overall survival [3]. Overall, pCR was found to have long-term benefits for patients, with the strongest association observed in triple-negative breast cancer (TNBC) and human epidermal growth receptor 2 (HER2)-positive breast cancer [9].

There has been a gap in the level of healthcare between developing and developed countries over the past 20 years, which has led to differences in drug availability. Although the NeoSphere study [10] published results as early as 2012 suggesting that pertuzumab combined with trastuzumab constituted an effective anti-HER2 treatment, pertuzumab was only approved for marketing in China in 2018. Such differences are bound to lead to variations in the prognosis of neoadjuvant chemotherapy for breast cancer. Thus, we reviewed data from a single institution in Asia to explore the long-term prognosis of neoadjuvant chemotherapy for breast cancer in developing countries.

## Materials & methods

### Patient characteristics

The study has been approved by the Ethics approval of Medical Ethics Committee of Affiliated Sir Run Run Shaw Hospital, Zhejiang University (SRRSH), (No. 20210910-30). In this study, 646 patients with early or locally advanced stage breast cancer, who underwent neoadjuvant therapy and surgery at the Department of Surgical Oncology, SRRSH, were retrospectively analyzed. They were selected from the 695 patients diagnosed with breast cancer at the department between February 25,1999 and August 22, 2018. In this cohort, all neoadjuvant chemotherapy/targeted therapy regimens and prescriptions were followed the NCCN guidelines [11]. The excluded 49 patients: [1] had received neoadjuvant endocrine therapy (n = 10), [2] progressed during neoadjuvant therapy (n = 15), [3] had occult breast cancer (n = 6), [4] presented male breast cancer (n = 2), [5] had deputy breast cancer (n = 2), [6] were primarily diagnosed with bone metastases (n = 5), or [7] had no available follow-up data (n = 9).

All the demographic variables were assembled into a database. All patients were periodically followed up after surgery. The final date of diagnosis was defined as baseline data. The primary endpoint of this study was 5-year RFS, which was specified as a comprehensive indicator of local relapse or distant metastasis of breast cancer, contralateral breast cancer, or death from any cause. The primary endpoint was determined retrospectively by two oncologists. The secondary endpoint was defined as 5-year OS from diagnosis to death from any cause or censoring surviving patients.

### Pathology review

All cases were diagnosed by core needle biopsy prior to treatment and estrogen receptor (ER), progesterone receptor (PR), HER2, and Ki-67 levels were determined by immunohistochemical staining. Our study used a Ki-67 cutoff of 25% for categorization. High Ki-67 expression was defined as Ki-67 > 25%.HER2-positivity was defined as having strong membrane staining patterns (3+) of the protein or gene amplification relative to the centromeric probe in ≥ 30% of tumor cells by fluorescence in situ hybridization (ERBB2/cep17 > 2.2) [12]. The ER positivity and PR positivity were defined as positive staining of tumor nuclei ≥ 10%. After the assessment of preoperative and postoperative pathological stages, the descending stage was defined as decreased postoperative pathological T stage or N stage compared with the preoperative stage. pCR is defined as the absence of invasive cancer in the breast and axillary nodes after surgery, following completion of neoadjuvant systemic therapy (i.e., ypT0/Tis ypN0 in the present American Joint Committee on Cancer (AJCC) staging system) [13]. The presence of lymphovascular space invasion (LVSI), extra-lymphatic dilatation, or pathologically positive lymph nodes were reported and evaluated by the pathologists from SRRSH.

### Statistical methods

The time interval from diagnosis to death or the last follow-up was calculated. The survival endpoints for overall survival (OS) and relapse-free survival (RFS) were defined as starting from the date of diagnosis using the published standardized criteria [14]. The Kaplan-Meier method and log-rank tests were used to compare the difference of 5-year OS and RFS. Multivariate Cox regression modeling for proportional hazards was employed to calculate the hazard ratio and 95% CI to assess the effect of factors on the OS and RFS. The factors included in the model were clinical stage, molecular subtype, Ki-67, surgery type, lymph node positive after neoadjuvant chemotherapy, lymph vascular invasion (LVSI), neoadjuvant chemotherapy response and NAC regimens. Multi-variate logistic regression model was simulated to predict pCR, which incorporating clinical stage, molecular subtype, ki-67, LVSI, treatment period and histology. The treatment period was divided into two time-groups according to the time of diagnosis of breast cancer: 1999–2009 and 2010–2018. ROC curve (Receiver Operating Characteristic) analysis was performed and an AUC (area under the curve) was calculated to evaluate the model. The significance of p value was set at *P* < 0.05. SPSS Statistics version 21 (IBM) was applied for statistical analyses and the survival graphs were plotted using GraphPad Prism 7.

## Results

### Patient and treatment characteristics

A total of 646 patients were included for the final analysis, with a median follow-up of 5.0 years (ranging from 0.4 to 18.0 years). The pretreatment characteristics, treatment strategies, as well as response and follow-up information of patients are summarized in Table 1. The median age of the cohort at diagnosis was 49 years old (ranging from 22 to 84).

After NAC, 118 patients (18.2%) in the cohort experienced pCR, whereas 528 (81.7%) exhibited residual disease. The pCR rate for the period of 2010 to 2018 was significantly higher than that for the previous decade. The majority of pCR patients were HER2-positive (41.5%), and Ki-67 > 25% (50.8%). In patients without pCR, the tumors were usually ER + and/or PR+, HER2-negative type (49.6%) and Ki-67 < 25% (58.1%). Whether or not patients achieved pCR, the clinicopathological characteristics showed statistically significant differences, including molecular typing, Ki-67, pathological lymph node staging (ypN), LVSI, surgical treatment, anti-HER2-targeted therapy, and treatment period (P < 0.05). About half of the HER2-positive patients received anti-HER2-targeted therapy (117:214) (Table 1). Only 6 patients in pCR patients were ypTis, which had little effect on the analysis of the whole cohort, so it was not reflected in Table 1.Consistent with common clinical observations, there were a number of cases of carcinoma in situ with focal invasion in non-PCR patients.

**Table 1:** Patient- and treatment-related characteristics

	Overall	Without pCR	pCR	P value
Age, years				0.789
≤ 40	115(82.2%)	95 (18.0%)	20 (16.9%)	
> 40	531(17.8%)	433 (82.0%)	98(83.1%)	
Menopausal status				0.221
Premenopausal	346 (53.6%)	289 (54.7%)	57 (48.3%)	
Postmenopausal	300(46.4%)	239(43.3%)	61(51.7%)	
Molecular subtype				**<0.001**
ER + and/or PR+, HER2-	294(45.5%)	262(49.6%)	32(27.1%)	
HER2+	214(33.1%)	165(31.3%)	49(41.5%)	
TNBC	138(21.4%)	101(19.1%)	37(31.4%)	
Ki-67				**<0.001**
≤ 25	347(53.7%)	307(58.1%)	40(33.9%)	
> 25	253(39.2%)	193(36.6%)	60(50.8%)	
unknown	46(7.1%)	28(5.3%)	18(15.3%)	
Histology				**<0.001**
Ductal	519(80.3%)	448(84.8%)	71(60.2%)	
Other	127(19.7%)	80(15.2%)	47(39.8%)	
Clinical stage				0.144
I	48(7.4%)	37(7.1%)	11(9.3%)	
II	374(57.9%)	299(56.6%)	75(63.6%)	
III	224(34.7%)	192(36.4%)	32(27.1%)	
ypN stage				**<0.001**
N0	473(73.2%)	355(67.2%)	118(100%)	
N+	173(26.8%)	173(32.8%)	0(0%)	
LVSI				**<0.001**
Absent	593(91.8%)	476(90.2%)	117(99.2%)	
Present	53(8.2%)	52(9.8%)	1(0.8%)	
Radiation therapy				0.522
No	224(34.7%)	180(34.1%)	44(37.3%)	
Yes	422(65.3%)	348(65.9%)	74(62.7%)	
Adjuvant hormonal therapy				0.035
No	404(62.5%)	320(60.6%)	84(71.2%)	
Yes	242(37.5%)	208(39.4%)	34(28.8%)	
Surgery type				0.074
Partial mastectomy	129(20.0%)	98(18.6%)	31(26.3%)	
Mastectomy	517(80.0%)	430(81.4%)	87(73.7%)	
Adjuvant targeted therapy				**<0.001**
No	529(81.9%)	448(84.8%)	81(68.6%)	
Yes	117(18.1%)	76(15.2%)	41(31.4%)	
NAC type				0.103
A + T	326(50.5%)	258(48.9%)	68(57.6%)	
Other	320(49.5%)	270(51.1%)	50(42.4%)	
Treatment period				**0.041**
1999–2009	291(45.0%)	248(47.0%)	43(36.4%)	
2010–2018	355(55.0%)	280(53.0%)	75(63.6%)	
Relapse				**<0.001**
No	484(74.9%)	375(71.0%)	109(92.4%)	
Yes	162(25.1%)	153(29.0%)	9(7.6%)	
Survival				**<0.001**
survival	448(69.3%)	342(64.8%)	106(89.8%)	
death	121(18.7%)	115(21.8%)	6(5.1%)	
lost to follow-up	77(11.9%)	71(13.4%)	6(5.1%)	

Abbreviations: pCR pathologic complete response; ER, estrogen receptor; PR; progesterone receptor; LVSI, lymphovascular space invasion; A + T, doxorubicin/epirubicin + paclitaxel/docetaxel; NAC, neoadjuvant chemotherapy

### Analysis of Relapse-Free Survival (RFS) and Overall Survival (OS)

**Fig. 1:**
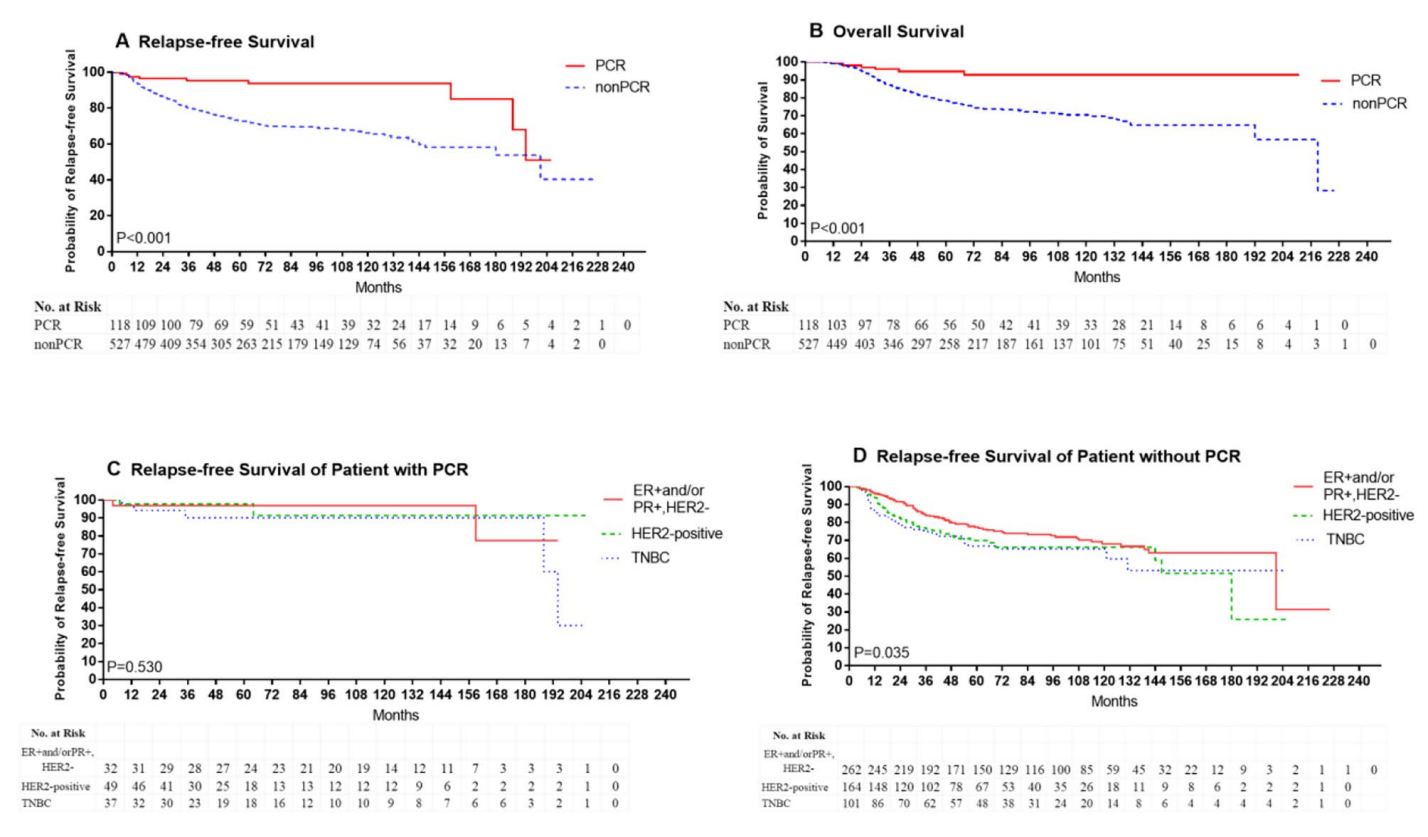
Kaplan–Meier plots for patient outcomes. The 5-year RFS rates (P < 0.001) (**A**) and 5-year OS rates (P < 0.001) (**B**) in patients with and without pCR. The 5-year RFS in subtype eithero of patient with pCR (**C**) and not achieving pCR (**D**)

Based on a median follow-up of 5.0 years (ranging from 0.4 to 18.0 years), the 5-year RFS rates were 95.3% and 72.7% in patients with and without pCR, respectively (P < 0.001) (Fig. 1A). Meanwhile, the 5-year OS rates were 94.6% versus 78.1% in patients with and without pCR, respectively (P < 0.001) (Fig. 1B).

Among patients who achieved pCR, the 5-year RFS was 96.9% for luminal, 97.9% for HER2-positive tumors, and 90.2% for TNBC (P = 0.530) (Fig. 1C). There was no difference in subtype either for those not achieving pCR either, with 5-year RFS rates ranging from 77.3% for ER + and/or PR+, HER2- type, 69.9% for HER2-positive tumors to 66.9% for TNBC tumors (P = 0.035) (Fig. 1D).

### Multivariate analysis

Factors independently predicting RFS in patients were HER2-positive subtype (hazard ratio, 1.906; 95% CI, 1.226–2.964; P = 0.004), TNBC (hazard ratio, 2.079; 95% CI, 1.280–3.378; P = 0.003), lymph node positive after neoadjuvant chemotherapy (hazard ratio, 2.939; 95% CI, 2.059–4.195; P < 0.001), pCR (hazard ratio, 0.396; 95% CI, 0.196–0.802; P = 0.010), and clinical stage III (hazard ratio, 2.950; 95% CI, 1.227–7.093; P = 0.016).

**Fig. 2:**
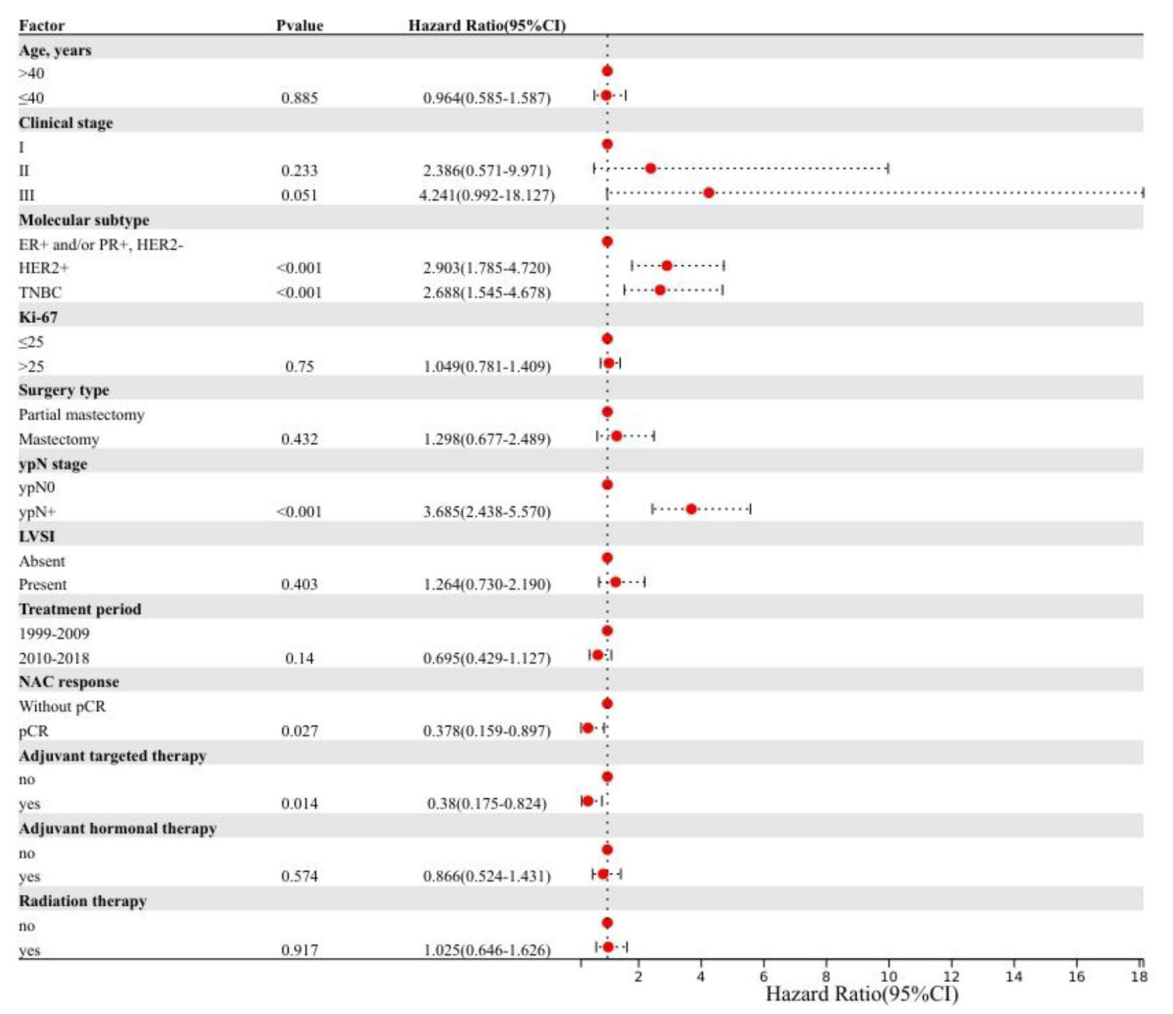
Forest plot of hazard ratios (HRs) and 95% confifidence intervals (CIs) of overall survival

Factors independently predicting OS in patients were HER2-positive subtype (hazard ratio, 2.903; 95% CI, 1.785–4.720; P < 0.001), TNBC (hazard ratio, 2.688; 95% CI, 1.545–4.678; P < 0.001), lymph node positive after neoadjuvant chemotherapy (hazard ratio, 3.685; 95% CI, 2.438–5.570; P < 0.001), clinical stage III (hazard ratio, 4.241; 95% CI, 0.992–18.127; P = 0.051), pCR (hazard ratio, 0.378; 95% CI, 0.159–0.897; P = 0.027), and adjuvant targeted therapy (hazard ratio,0.380; 95% CI, 0.175–0.824; P = 0.014) (Table 2; Fig. 2).

**Table 2:** Multivariate Cox Analysis of relapse-free survival and overall survival

	OS				RFS		
Factor	HR	95% CI	*P*		HR	95% CI	*P*
**Age, years**							
> 40	1				1		
≤ 40	0.964	0.585–1.587	0.885		1.287	0.872-1.900	0.203
**Clinical stage**							
I	1				1		
II	2.386	0.571–9.971	0.233		1.313	0.560–3.077	0.531
III	4.241	0.992–18.127	**0.051**		2.950	1.227–7.093	**0.016**
**Molecular subtype**							
ER + and/or PR+, HER2-	1				1		
HER2+	2.903	1.785–4.720	**< 0.001**		1.906	1.226–2.964	**0.004**
TNBC	2.688	1.545–4.678	**< 0.001**		2.079	1.280–3.378	**0.003**
**Ki-67**							
≤ 25	1				1		
> 25	1.049	0.781–1.409	0.750		0.967	0.749–1.247	0.794
**Surgery type**							
Partial mastectomy	1				1		
Mastectomy	1.298	0.677–2.489	0.432		1.050	0.646–1.704	0.843
**ypN stage**							
ypN0	1				1		
ypN+	3.685	2.438–5.570	**< 0.001**		2.939	2.059–4.195	**< 0.001**
**LVSI**							
Absent	1				1		
Present	1.264	0.730–2.190	0.403		1.316	0.812–2.133	0.266
**Treatment period**							
1999–2009	1				1		
2010–2018	0.695	0.429–1.127	0.140		0.930	0.650–1.429	0.741
**NAC response**							
Without pCR	1				1		
pCR	0.378	0.159–0.897	**0.027**		0.396	0.196–0.802	**0.010**
**Adjuvant targeted therapy**							
no	1				1		
yes	0.380	0.175–0.824	**0.014**		0.554	0.301–1.020	0.058
**Adjuvant hormonal therapy**							
no	1				1		
yes	0.866	0.524–1.431	0.574		1.088	0.709–1.670	0.699
**Radiation therapy**							
no	1				1		
yes	1.025	0.646–1.626	0.917		1.058	0.704–1.592	0.785

Abbreviations: RFS, relapse-free survival; ER, estrogen receptor; HR, hazard ratio; OS, overall survival; PR, progesterone receptor; LVSI, lymphovascular space invasion

### Multi-variate logistic regression model to predict pCR

**Fig. 3:**
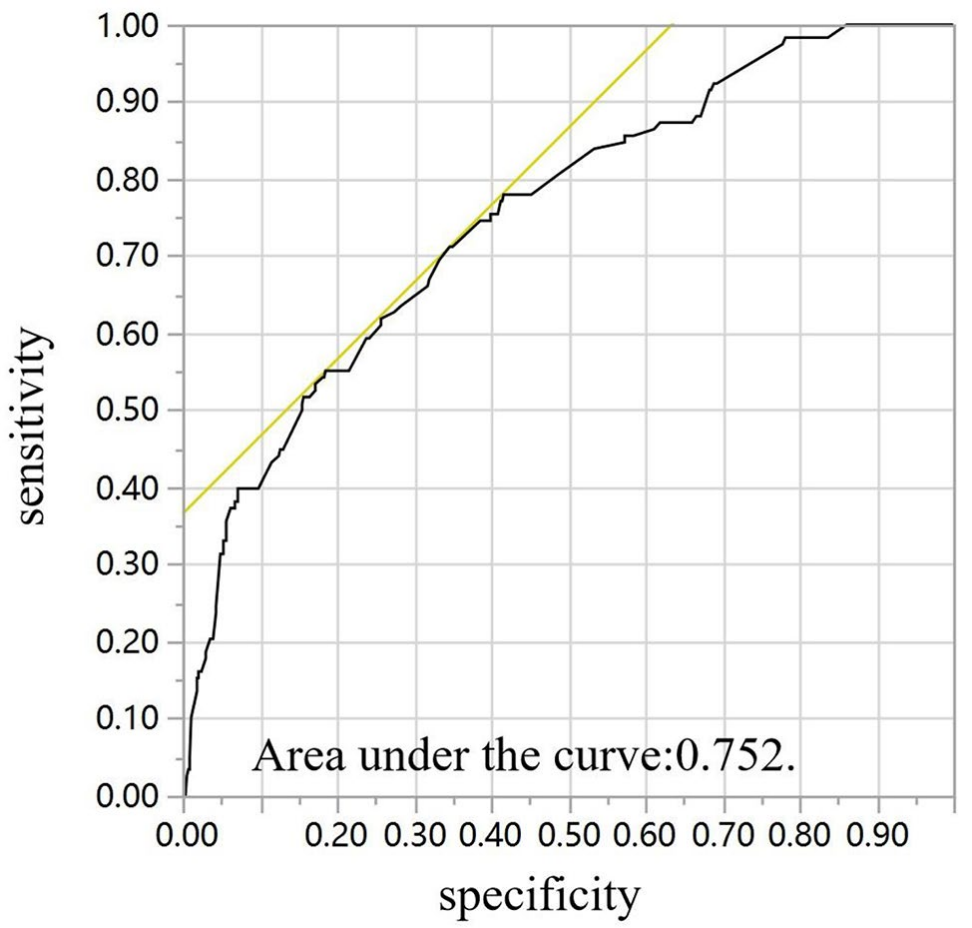
Receiver operating characteristic (ROC) curve for the multivariate predictive nomogram of the pathologic complete response rate after neoadjuvant chemotherapy in breast cancer patients

Multi-variate logistic regression model was simulated to was developed to predict the breast cancer pCR rate after NAC, according to clinical stage, molecular subtype, ki-67, LVSI, treatment period and histology. In the ROC curve analysis, the AUC of the nomogram was 0.752 (Table 3; Fig.3).

**Table 3:** Multivariable logistic regression model predicting pCR

	Odds Ratio(95% CI)	P value
Age(per unit)	1.21(0.68–2.15)	0.505
Molecular subtype		
ER + and/or PR+, HER2-	Reference	
HER2+	1.95(1.13–3.36)	0.016
TNBC	2.01(1.11–3.65)	0.022
Ki-67		
≤ 25	Reference	
> 25	1.64(0.99–2.68)	0.051
unknown	NA	
Histology		
Ductal	0.30(0.19–0.48)	< 0.0001
Other	Reference	
Clinical stage		
I	Reference	
II	0.82(0.37–1.80)	0.617
III	0.41(0.17–0.98)	0.044
LVSI		
Absent	Reference	
Present	0.09(0.01–0.66)	0.018
Treatment period		
1999–2009	Reference	
2010–2018	1.91(1.17–3.13)	0.009

## Discussion

In this study, we found that the pCR rate of breast cancer after neoadjuvant chemotherapy in our center was similar to the international level in the past 20 years. The rate of pCR in our cohort was 18.2%, which was close to most previous findings. A large meta-analysis [15] included a total of 52 studies and 27,895 women treated with the neoadjuvant approach. They showed an overall pCR rate of 21.1% (range: 10.1–74.2%). The highest rates of pCR were seen in HER2-positive tumors at 36.4% (range: 17.5–74.2%) and TNBC at 32.6% (range: 20.3–62.2%), and the hormone receptor positive (HR+)/HER2- tumors were the lowest at 9.3% (range: 5.5–31.3%). In particular, consistent with the above meta-analysis, HR+/HER2- patients were also associated with lower pCR rates than TNBC and HER2-positive subtypes [16]. Another meta-analysis showed that HER2-positive breast cancer also had an advantage in axillary pCR rate [17].

In a pooled analysis of 12 clinical trials by Cortazar et al. (2014), the authors demonstrated that pCR was associated with improved event-free survival (EFS), while the association between the magnitude of treatment-induced pCR change and corresponding improvement in EFS could not be established [9]. Nevertheless, the 3-year outcomes of the I-SPY2 trial showed that, regardless of subtype and treatment regimen, achieving pCR after neoadjuvant therapy including 9 novel therapeutic combinations implied an approximately 80% reduction in relapse rate [18]. As expected, Spring et al. [15] demonstrated the very strong correlation of pCR with EFS. The achievement of pCR following NAC is associated with significant better EFS and OS, particularly for triple-negative and HER2-positive breast cancer. Another meta-analysis also suggested that pCR in HER2-positive breast cancer is more likely [19]. The results in our study also suggest that HER2-positive and triple-negative breast cancers were sensitive to neoadjuvant therapy, however, there is no statistically significant survival advantage after neoadjuvant therapy compared with luminal subtype. In our study, the total treatment efficacy was comparable in the 5-year RFS rates of 95.3% and 72.7% in patients with and without pCR (P < 0.001) as compared with the studies of Spring et al. [15], where the pCR patients had a 5-year EFS of 88% (95% PI: 85-91%) while those without pCR exhibited a 5-year EFS of 67% (95% PI: 63-71%). In multivariate analysis, pCR indicated better OS (hazard ratio, 0.378; 95% CI, 0.159–0.897; P = 0.027) and RFS (hazard ratio, 0.396; 95% CI, 0.196–0.802; P = 0.010).

Furthermore, the HER2-positive breast cancer type does not present an advantage for prognosis, which is different from the results of many other studies [20]. As mentioned above, almost half of HER2-positive patients receive anti-HER2-targeted neoadjuvant therapy (117:214) (Table 1). For economic reasons, targeted therapies were still not widely used in China in early 2000s. The NeoSphere study [10] results indicated that pertuzumab combination therapy and trastuzumab plus docetaxel significantly increased pCR and 5-year DFS. The KATHERINE study [21], which further investigated the effects of adjuvant therapy after neoadjuvant targeted therapy on HER2-positive breast cancer, suggested that adjuvant T-DM1 can benefit patients whose neoadjuvant targeted therapy does not achieve pCR. While the case data in our paper was from the period of 1999 to 2018, pertuzumab was only applied in China at the end of 2018. In the past five years, targeted tumor therapy has been almost fully implemented in China.

The factors predicting non-pCR of NAC are still not clear. The most common prognostic factors for patients without successful pCR are residual cancer burden [22], Ki-67 [23], and tumor-infiltrating lymphocytes [24, 25]. In this study, we find LVSI, Ki-67, HER2 subtype were significant associated with pCR of patients who received NAC. However, the authors repeatedly wondered whether the prognostic indicator factors of residual cancer burden for patients without pCR could be simplified in the analysis. Neoadjuvant chemotherapy is an autologous drug sensitivity test and those sensitive to chemotherapy patients could have higher probability of achieving pCR. PCR to neoadjuvant chemotherapy is associated with favorable long-term outcomes.

The response to pre-surgery treatment was shown to have the ability to predict the subsequent outcome of breast cancer [2, 26, 27], which makes pCR a valuable intermediate endpoint for evaluating the efficacy of preoperative treatment regimens. The significance of neoadjuvant therapy is to improves breast-conserving surgery success rates due to tumor downstaging, and pCR to neoadjuvant chemotherapy is associated with favorable long-term outcomes. In other words, patients without pCR require further postoperative adjuvant intensive therapy to improve survival, which is currently the most important value of pCR: chemotherapy sensitivity screening. The CREATE-X [28] and KATHERINE [21] trials have transformed clinical practice by showing that capecitabine and T-DM1, respectively, significantly improve outcomes in TNBC and HER2-positive breast cancer patients who did not achieve pCR after neoadjuvant chemotherapy. To some extent, patients without pCR can benefit from additional post-surgical treatments, while the determination of adjuvant therapy intensity is the focus of future research.

The present study identified the following prognostic factors of breast cancer outcomes for patients: HER2-positive subtype, TNBC, pCR, Ki-67 > 25, higher pathologic nodal stage, later clinical staging, and treatment period. The Ki-67 protein has been reported to be an independent predictor of pCR, overall survival, and distant disease-free survival [29], which is consistent with the research results of this paper. The treatment period has an impact on OS, as the level of treatment in the past 10 years has significantly improved compared with that before 2010 due to the progress of international communication and the standardization of treatment in China. Studies on predictors without pCR after neoadjuvant chemotherapy for triple-negative breast cancer have demonstrated that pathological lymph node positivity and LVSI predict worse outcomes [30, 31]. In this study, however, LVSI was not a meaningful prognostic factor (hazard ratio, 1.241; 95% CI, 0.767–2.006; P = 0.379). The study cohort was sourced from a single facility in a tertiary level A hospital in a certain developing country, therefore the patient population may not be representative of various institutions in different developing countries. The regulatory variables used for multivariate analysis may be incomplete, and the absence of certain variables, including tumor infiltrating lymphocytes, p53 status, necrosis, etc., for which no data has been collected, may affect the results.

There was notable limitation in our study. It was conducted at a single center and was retrospective, which creates a susceptibility to selection bias. However, we studied a larger number of patients, and the choice of chemotherapy regimens followed the same principles. Therefore, we ‘re doing a multi-center data analysis.

## Conclusions

This study concluded that the post-neoadjuvant chemotherapy pCR rate for breast cancer in our center has kept pace with the international level in the past 20 years. The correlation between pCR and better outcomes was consistent. Following NAC, HER2-positive subtype and TNBC molecular subtype had higher pCR rates.

### Electronic supplementary material

Below is the link to the electronic supplementary material.

Supplementary Material 1

## Data Availability

All data generated or analysed during this study are included in this published article [and its supplementary information files].
